# Contextual spatial modelling in the horizontal and vertical domains

**DOI:** 10.1038/s41598-022-13514-5

**Published:** 2022-06-09

**Authors:** Tobias Rentschler, Martin Bartelheim, Thorsten Behrens, Marta Díaz-Zorita Bonilla, Sandra Teuber, Thomas Scholten, Karsten Schmidt

**Affiliations:** 1 SFB 1070 ResourceCultures, University of Tübingen, 72074 Tübingen, Germany; 2 Cluster of Excellence Machine Learning: New Perspectives for Science, University of Tübingen, 72076 Tübingen, Germany; 3 eScience-Center, University of Tübingen, 72074 Tübingen, Germany; 4 Department of Geosciences, Chair of Soil Science and Geomorphology, University of Tübingen, 72070 Tübingen, Germany; 5 Department of Pre- and Protohistory and Medieval and Post-Medieval Archaeology, University of Tübingen, 72070 Tübingen, Germany; 6 Soilution GbR, Soil and Spatial Data Science, 06484 Quedlinburg, Germany; 7 Swiss Competence Center for Soils (KOBO), Data Science, BFH-HAFL, CH-3052 Zollikofen, Switzerland

**Keywords:** Geomorphology, Environmental impact, Computer science

## Abstract

Multi-scale contextual modelling is an important toolset for environmental mapping. It accounts for spatial dependence by using covariates on multiple spatial scales and incorporates spatial context and structural dependence to environmental properties into machine learning models. For spatial soil modelling, three relevant scales or ranges of scale exist: quasi-local soil formation processes that are independent of the spatial context, short-range catenary processes, and long-range processes related to climate and large-scale terrain settings. Recent studies investigated the spatial dependence of topsoil properties only. We hypothesize that soil properties within a soil profile were formed due to specific interactions between different features and scales of the spatial context, and that there are depth gradients in spatial and structural dependencies. The results showed that for topsoil, features at small to intermediate scales do not increase model accuracy, whereas large scales increase model accuracy. In contrast, subsoil models benefit from all scales—small, intermediate, and large. Based on the differences in relevance, we conclude that the relevant ranges of scales do not only differ in the horizontal domain, but also in the vertical domain across the soil profile. This clearly demonstrates the impact of contextual spatial modelling on 3D soil mapping.

## Introduction

Soils are crucial for agriculture, forestry, biodiversity, biofuels production, and global carbon cycling^1,2^. The growing world population requires changes in food production towards sustainability through policies and management practices^1,3^. Spatial knowledge of soil properties and processes can support management practices to increase the productivity which depends on water content, nutrient availability, soil acidity, and other soil quality indicators^1^. Relevant data can be obtained from sophisticated soil models and spatial predictions based on machine learning. Such predictions of soil properties are based on two fundamental paradigms: the state factor equation (Eq. 1)^4^ and the universal model of spatial variation (Eq. 2)^5^. The state factor equation formalises the concept of soil forming factors^6^:

1 $$S = f\left( {cl, o,r,p,t, \ldots } \right)$$

where *S* is a soil property at a certain location that develops as a non-linear function *f* of the soil forming factors climate (*cl*), organisms (*o*), relief (*r*), parent material (*p*), time (*t*), and other potentially unknown factors (⋯), which may include other soil properties^7^, spatial location^7^, and spatial context^8^. Thus, the universal model of spatial variation includes the deterministic state factor equation as well as a stochastic part of (apparently) random variation:

2 $$Z\left( s \right) = Z^{*} \left( s \right) + \varepsilon^{\prime}\left( s \right) + \varepsilon^{\prime}$$

where *Z*(*s*) is the soil property, Z*(*s*) the deterministic component, i.e., the soil forming factors from Eq. (1), *ε*′(*s*) the stochastic component of (apparently) random variation, and *ε*′ random noise. While Z*(*s*) can be modelled with machine learning methods by correlating the outcome with the independent environmental covariates of the state factor equation, the stochastic component *ε*′(*s*) is often modelled with geostatistical methods such as kriging^9,10^ or Bayesian approaches^11,12^. However, with multi-scale environmental covariates Behrens et al.^13^ showed that *ε*′(*s*) can actually be treated as part of Z*(*s*) and mention short-range catenary and long-range teleconnected aeolian systems as example because both depend on relief (*r*) and climate (*cl*) but on higher hierarchical levels^14^.

Recent studies incorporating multi-scale environmental covariates into machine learning models showed increasing prediction accuracy, parsimony, and computational efficiency^8,15–18^. Further, some of the referenced studies enable and foster complex geoscientific and pedological interpretations with respect to soil forming processes^13,18–21^. The higher accuracy of the multi-scale approaches compared to approaches without accounting for the spatial context is related to the spatial variation and the interactions of soil properties and soil forming processes that are effective on multiple scales at the same time as well as over the temporal dimension. Consequently, machine learning models in combination with feature engineering techniques can account for contextual information *ε*′(*s*) that reflects interacting, hierarchical, and scale dependent soil forming processes in the horizontal domain. Recent studies focused on topsoil only (mostly 0–30 cm)^14,20,22^, but neglected subsoil (> 30 cm). However, the whole soil continuum is relevant for environmental processes and several studies have shown that the factors of soil formation are also effective in the vertical domain, for example climate (*cl*)^23,24^, vegetation (*o*)^23–25^, relief (*r*)^19,24,26,27^, parent material (*p*)^22,28^, and other environmental properties (⋯)^28,29^. Therefore, we investigated the influence of spatial context in modelling the horizontal and the vertical domains and hypothesised that soil forming processes in subsoil relate differently to the spatial context compared to topsoil.

Behrens et al.^20^ presented two approaches to identify the appropriate scale space for spatial modelling: using the empirical variogram to determine the range of geostatistical models (kriging), and using machine learning model sequences where coarser scales are added or dropped sequentially from the covariate space. Our objectives with this paper were (i) to test if different spatial scales may be relevant for the vertical domain, and (ii) to interpret and discuss how and why different spatial scales may influence soil properties and soil forming processes across a soil profile. To do so, we closely followed the approaches of multi-scale contextual spatial modelling to derive multi-scale terrain derivatives with the Gaussian pyramid^17,18,20^, and the empirical variogram. We created a soil dataset of 130 soil profiles with up to 5 depth increments depending on the local soil depth. This dataset includes measurements of soil quality indicators, soil properties derived from pedo-transfer functions, and a soil quality index.

## Material and methods

### Study area

**Figure 1:**
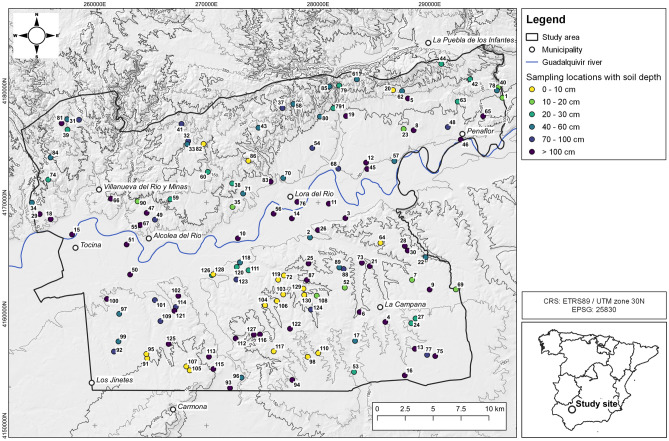
Map of the study area in Andalusia, Spain, with soil sampling locations and the corresponding soil depth (cm) of each profile according to the sampling design (Hill shade and contour lines derived from the digital elevation model used in this study by CNIG with QGIS^34^. Administrative map data for the lower right panel provided by gadm.org, https://gadm.org/license.html).

The study area of 1000 km^2^ is located in the Middle Guadalquivir basin, Andalusia, Spain, 50 km NE of Seville (Fig. 1). The geologic setting separates the landscape in three main parts: (i) the Sierra Morena mountain range in the North with Palaeozoic granite, gneiss, and slate, (ii) the Guadalquivir flood plain with Pleistocene marl, calcarenite, coarse sand, and Holocene sands, and loams, and (iii) Neogene terraces of coarse gravel and cobble with sands and loams in the South^30–32^. The slopes are typically 50–1000 m long. The study area is a heterogenous agricultural landscape with arable land, citrus, and olive plantations, pastures, and Dehesa, an agro-sylvo-pastoral system. According to the USDA soil taxonomy the predominant soil types are Alfisols, Entisols, Inceptisols, and Vertisols^33^.

### Soil data and environmental covariates

Soil samples were taken at 130 stratified random locations in October 2018. The strata are combinations of four geomorphic positions^35^ (flat, footslope, slope and shoulder) and the CORINE Land Cover level 2 classes arable land, permanent crops, pastures, forest, and shrub and/or herbaceous vegetation associations, which are the most common land cover classes in the study area. The point density of the sampled locations is proportional to the stratum area with a minimum of 3 samples for the smallest stratum. At each location up to five samples were taken with an auger depending on the soil thickness and bulked from 3 replicates. The sampled increments were 0–10, 10–20, 20–30, 40–60, and 70–100 cm. We defined the sampled increments from 0 to 30 cm as topsoil and 30–100 cm as subsoil.

For lab analysis, the samples were dried at 40 °C for 24 h, root fragments were removed, sieved (< 2 mm), and ground. The spectra of all 506 samples were measured with a Tensor II (Bruker Optics, Ettlingen, Germany) for reflectance in the NIR spectrum (833–3500 nm, i.e. 2860–12,000 cm^−1^ with a resolution of 4 cm^−1^) and a GladiATR (Pike Technologies, Madison, WI, USA) in the MIR spectrum (2270–25,000, i.e. 400–4400 cm^−1^ with a resolution of 2 cm^−1^). Due to the overlapping spectra, we cut the spectra at 2500 nm and kept the spectra from 833 to 2500 nm measured with the Tensor II and from 2500 to 25,000 nm measured with the GladiATR. A subset of 97 samples representative for the strata was analysed for soil organic carbon (SOC) with a Vario EL III (Elementar, Hanau, Germany), for soil acidity (pH in KCl solution; pH_KCl_) with a ProfiLine pH 3310 and a SenTix 81 electrode (WTW, Weilheim, Germany), and for grain size fractions clay (< 2 µm), silt (2–50 µm) and sand (50–2000 µm) with a SediGraph III (Micromeritics, Norcross, GA, USA). The 97 spectra were used as dependent variables to train partial least squares regression models and to make predictions for the remaining 409 samples^36^. Since pre-processing of the spectra^37^ did not improve model performance, the raw spectra were used. The root mean squared errors of these models were 0.5% for SOC content, 0.4 for pH, 4% for clay, 5% for silt, and 5% for sand content. The predictions of SOC content ranged from 0.01 to 3.61%, the pH from 3.00 to 12.11, the clay content from 0.01 to 73.94%, the silt content from 0.01 to 53.54%, and the sand content from 0.01 to 101.55%. The sum of the texture fractions ranged from 90 to 110% with a standard deviation of σ = 3.25%.

We derived the cation exchange capacity (CEC in cmol kg^−1^) based on a pedo-transfer function developed specifically for Spain^38^ and the water content at field capacity (θ_FC_ in cm^3^ cm^−3^) using a pedo-transfer function developed for Europe^39^. The root mean square errors (RMSE) of these pedo-transfer functions were reported to be 0.06 cm^3^ cm^−3^ and 0.73 cmol kg^−1^, respectively. Subsequently, we calculated a soil quality rating (SQR) to represent the yield potential of soils for agricultural crops based on the CEC, pH, and θ_FC_ values^40,41^.

### Variography

The variogram is a geostatistical model^42^. Empirical data is used to describe the degree of spatial autocorrelation between pairs of point measurements of environmental properties and to develop a theoretical model, which can be used for spatial modelling using kriging. The variogram consists of a set of parameters: nugget, sill, lag, and range. The nugget is the y-intercept and describes the variability at the smallest scale of the data related to noise and errors. The sill represents the maximum variability between point pairs. The lag is the radius in which point pairs are built. The spatial support that the lag corresponds to in this study is twice the spatial support of the pixel size of the downscaled Gaussian octave (see next section). The range is the maximum distance up to which the data is spatially autocorrelated. Therefore, the range is an indicator of the maximum scale of spatial context for environmental modelling^16,20,22^.

### Gaussian pyramid mixed scaling

Gaussian mixed scaling^17,18^ is a method to derive multi-scale terrain derivatives to incorporate the spatial contextual information of a landscape in a machine learning model. Gaussian mixed scaling is based on Gaussian filtering and down-sampling^43^ and decomposes the scales of environmental covariates^17^. In the Gaussian pyramid, each down-sampling step removes every second column and row of the digital elevation model (DEM) of the previous step. Possible associated artifacts are minimised with a Gaussian filter before each downscaling step. The outcome of each step is called an octave. Finally, all octaves are up-sampled with an inverse scaling procedure to the original resolution of the DEM to enable subsequent data manipulation on the same resolution.

**Figure 2:**
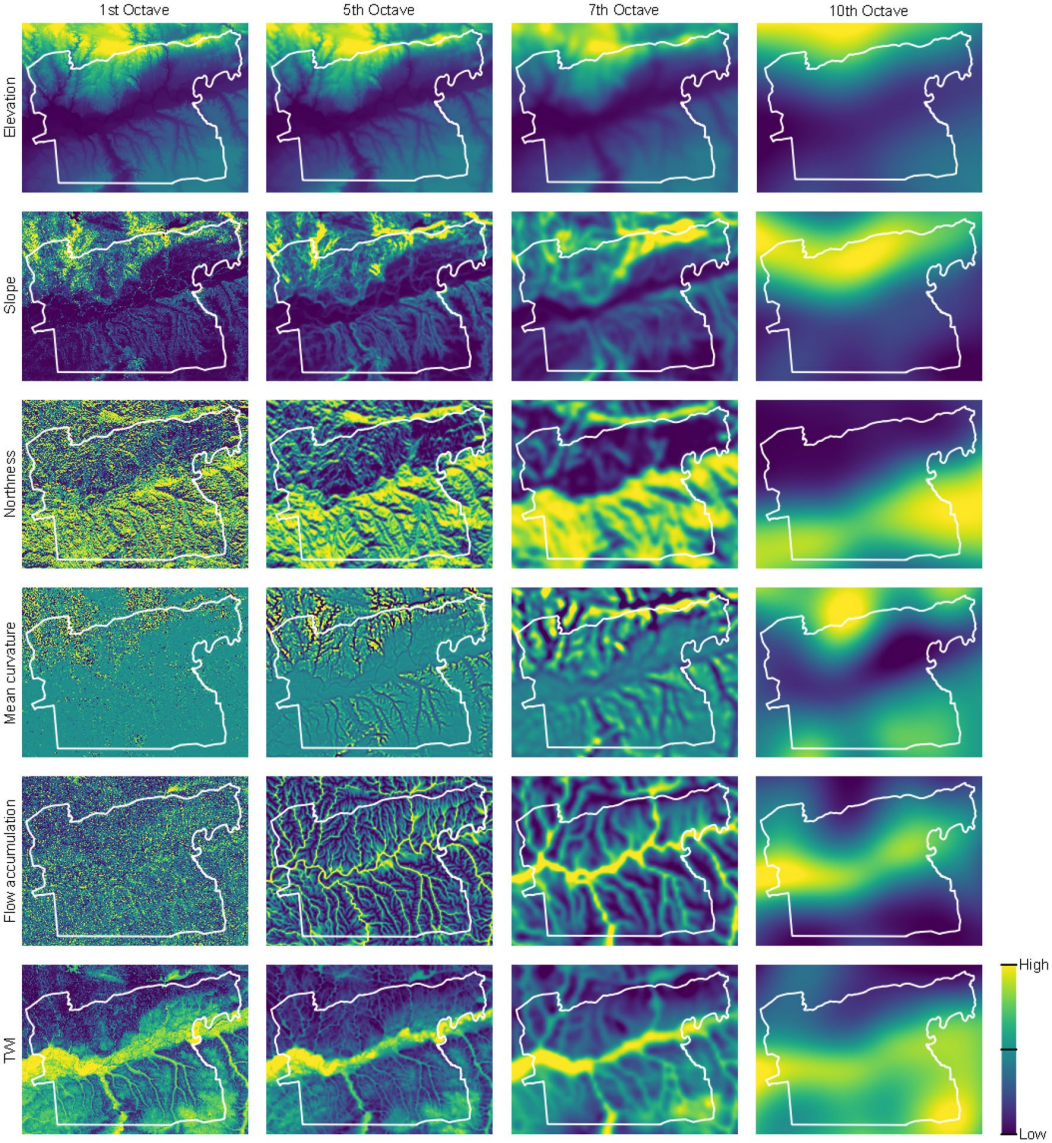
Selection of environmental covariates showing elevation, slope, northness, mean curvature, flow accumulation, and the topographic wetness index (TWI) at the 1st (10 m scale), 5th (160 m), 7th (640 m), and 10th octave (5120 m).

In mixed scaling, the DEM is downscaled. Then the terrain covariates are derived from each octave and are subsequently upscaled. Mixed scaling has demonstrated to be more accurate than scaling of the derived terrain covariates while providing less artefacts and a better basis for interpretation^18^. We used the following terrain covariates based on Zevenbergen & Thorne’s equations^44^ derived from the DEM of 5 m resolution: elevation, slope, sine transformed aspect, cosine transformed aspect, average curvature, profile curvature, planform curvature, flow accumulation, and topographic wetness index (Fig. 2) and calculated 11 octaves and 11 intermediate levels^18^ with corresponding cell sizes of the Gaussian scale space: 10, 20, 40, 80, 160, 320, 640, 1280, 2560, 5120, and 10,240 m. These covariates are the deterministic part Z*(*s*) and also reflect the seemingly random variation e′(*s*) in the universal model of spatial variation as well^13^. In respect to the average length of slopes in the study area, the 1st to 4th octaves (10–80 m) describe small scale properties of the terrain. The 5th to 8th octaves (160–1280 m) represent features of the terrain on the intermediate scale and are related to catenary soil forming processes, such as erosion, sediment transport, and reallocation. The 9th to 11th octaves (2560–10,240 m) describe large scale supraregional features of the landscape, i.e. the geomorphic signature of the landscape^17^, such as the mountain range in the north and the flood plain of the Guadalquivir river in the centre of the study area.

### Machine learning and validation

Machine learning methods have proven to be able to extract relationships between soil data and independent environmental covariates. Amongst others, random forests are applied often for spatial soil modelling in 3D^18,19,28,45^. Random forests is an ensemble of classification and regression trees^46,47^. In a decision tree binary splits are used recursively to homogenize the predictor variables in relation to the dependent variable, thus minimizing node impurity. Random forests use a bootstrapping approach, where a random set of predictor variables is tested at each split of a tree. The final regression model results from averaging all outputs of the decision tree ensemble. Random forests are relatively robust against overfitting and multi-collinearity, and provide a tool which facilitates interpretations of the models^47^. In this study, we used the random forest implementation in R^48–50^. For model evaluation, we used Pearson’s correlation coefficient (R^2^). The models were trained with grid tuning in search for the optimal model configuration^51^ and validated with ten times repeated ten-fold cross-validation using the caret package^52^.

We applied additive and subtractive multi-scale models^20^ to analyse the influence of the increasing information horizon^53^. We compared and analysed repeated model training and sequential adding of octaves representing larger scales to the training set as well as sequential subtraction (dropping) of smaller octaves from the complete set of covariates. The additive model sequence starts with the covariates at the original resolution of the DEM and subsequently adds the covariates of the next higher octave to the covariate space for model training. For the subtractive model sequence, the covariates of the smallest octaves of the covariate space are dropped sequentially. In this way a certain scale is represented by two models that contain either all smaller octaves (additive model sequence) or all larger octaves (subtractive model sequence) of the complete covariate space. With this approach increases or decreases of R^2^ values of the model sequences indicate which octaves and scales are more relevant for the model training^20^. For better visual analysis, the model results are smoothed with locally estimated scatterplot smoothing (loess^48^). In total, we trained and validated 920 models (11 octaves with intermediate levels for four soil properties in five depths with additive and subtractive modelling, respectively).

## Results

### Variography

**Figure 3:**
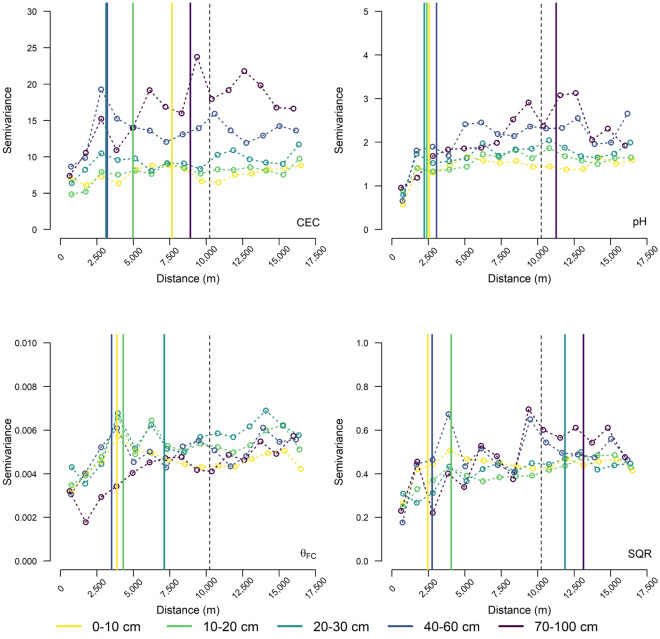
Empirical isotropic variograms for cation exchange capacity (CEC), soil acidity (pH), and water content at field capacity (θ_FC_), and the soil quality rating (SQR) at the five soil depths (0–10, 10–20, 20–30, 40–60, and 70–100 cm). The solid vertical lines indicate the range of the theoretical spherical isotropic variograms for each depth increment and the dashed vertical line indicates the maximum range of the contextual machine learning models (range of θ_FC_ at 70–100 cm was 29,000 m and outside the plot boundaries).

The empirical variograms for CEC, pH, θ_FC_, and SQR at the five soil depths (Fig. 3) show an overall increase of semivariance with increasing distance. The largest increase in semivariance was below a distance of 2500 m corresponding to the ninth octave. The ranges of the (theoretical) spherical isotropic variograms^20^ varied between 3000 and 9000 m for CEC, 2500–11,000 m for pH, 3500–29,000 m for θ_FC_, and 2500–13,000 m for SQR (Fig. 3). The largest range of the spherical isotropic variograms of 13,000 m was near the 11th octave (scale 10,240 m) which was chosen as highest octave. However, in some cases the empirical variograms showed increasing semivariance beyond the range of the theoretical variogram as also reported by Behrens et al.^20^.

### Machine learning

**Figure 4:**
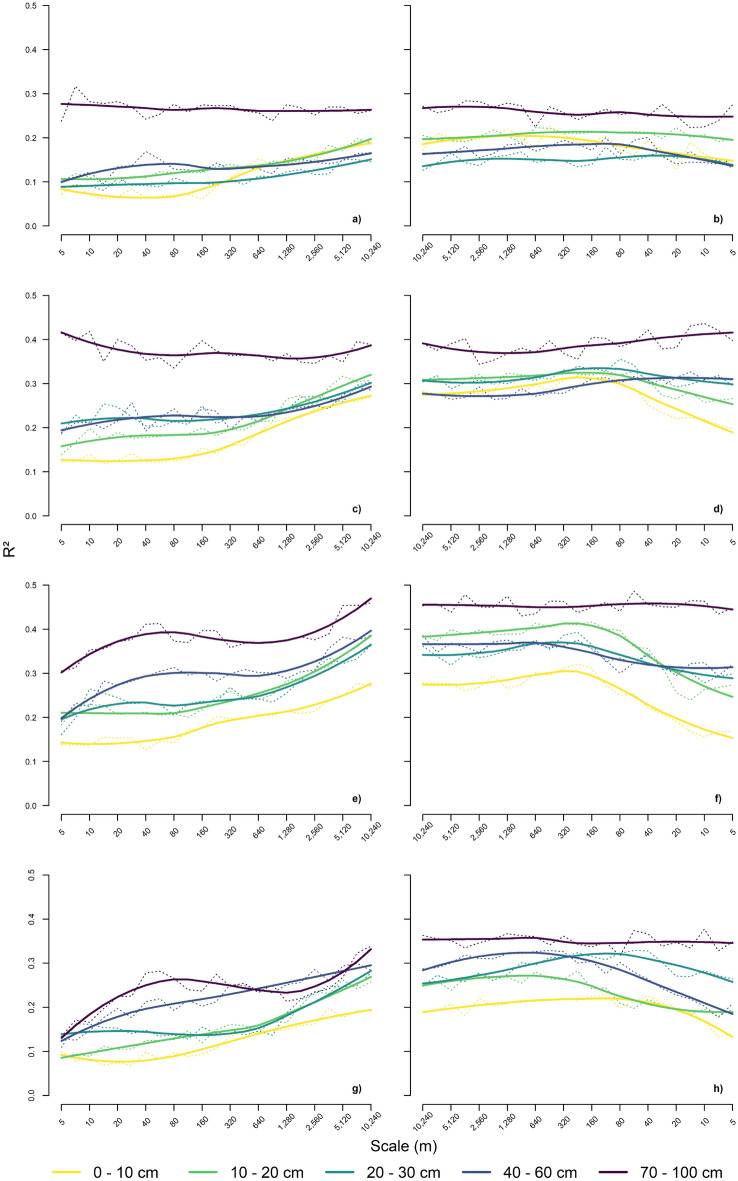
Results of the contextual machine learning models for cation exchange capacity (CEC; first row), soil acidity (pH; second row), water content at field capacity (θ_FC_; third row) and the soil quality rating (SQR; fourth row) at the five soil depths (0–10, 10–20, 20–30, 40–60, and 70–100 cm). The left panels (a, c, e, and g) show the additive models, and the right panels (b, d, f, and h) show the subtractive models. The correlation of the model predictions with the measured values are represented by the dotted lines, and smoothed trends are represented by solid lines (see Supplementary Tab. S1 for details).

For most of the model sequences with the soil quality indicators (CEC, pH, θ_FC,_ and SQR; Fig. 4; Supplementary Tab. S1) at five depth increments, successive addition (left panels in Fig. 4) of coarser scales of the covariates generally increased the model accuracy (R^2^), while successive (right panels in Fig. 4) removal of finer scales generally decreased model accuracy at distinct scales. Both, increase and decrease were not continuous in most model sequences.

In some model sequences with the additive approach, the model accuracies started to increase at a scale of 80–160 m for CEC, pH, and θ_FC_ (Fig. 4) in topsoil. While this was also the case for CEC and pH in subsoil in 40–60 cm depth, the increase for θ_FC_ starts with the first octave similarly to the increment of 70–100 cm. At the increment 70–100 cm models for CEC showed a marginal decrease over all scales and models for pH show a slight decrease. The subtractive model sequences for topsoil and 40–60 cm mostly showed a decrease in R^2^ after removal of the scales larger than 160–320 m. The model sequences for CEC and θ_FC_ at 70–100 cm showed marginal decreases over the whole sequence, whereas the sequence for pH showed a slight increase in R^2^. The increase of the additive model sequence for SQR in 0–10 cm (Fig. 4) started at a scale of 40 m, in the second and third depth increment at 320–640 m, the model sequence for SQR in 40–60 cm showed a continuous increase across all scales, and in 70–100 cm an increase to 80 m with a plateau from 160 to 640 m, and an additional increase from 1280 m onwards. In the subtractive model sequence, the decreases in R^2^ start with the corresponding scales for the depth increments from 0 to 60 cm, whereas there is no decrease for the sequence for the 70–100 cm increment.

Compared to models that used the terrain derivatives of the original resolution of the DEM of 5 m only, the model sequences mostly improved predictive accuracy of the models for all soil properties, CEC, pH, θ_FC_, and SQR. The increase ranged from 10% for CEC (0–60 cm) to 20 in SQR (70–100 cm) in the explained variance. Two models, CEC, and pH at 70–100 cm, did not benefit from increasing scales.

## Discussion

The a priori approximation of the variogram showed that the strongest increase of semivariance was below 2500 m. This indicates that the strongest spatial relation for most soil properties and depth increments is found below 2500 m and the corresponding 9th octave. The increase of semivariance above this range was covered with two additional octaves to account for potential spatial dependencies beyond the range of the theoretical variogram^20^. The ranges of the variograms were different for the four soil properties as well as the five depth increments.

For all four soil properties, the samples from 70 to 100 cm had the largest ranges (9000–29,000 m), whereas models for the increments 0–60 cm had smaller ranges. The larger ranges for the lower depth interval may reflect the large-scale variation of the regional geology, which varies over distances of several ten kilometres. This points to soil formation processes like weathering of Palaeozoic bedrock in the Sierra Morena mountain range, and large-scale translocation processes such as sediment transport by water and river meandering in the Guadalquivir flood plain^54^ formed mainly during the Pleistocene, and terrace formation during the Neogene^55^. Further, large-scale climate changes during the Holocene^56^ might have altered the weathering conditions of the whole area and is reflected in stable soil properties like soil texture that controls water holding capacity and CEC to a large extend^57^, and therefore, soil quality, but also soil acidity due to the presence of carbonates in Neogene sediments^32^. However, a trend for the maximum range of the variogram in respect to soil depth, i.e., an in-situ continuously increasing range with increasing soil depth, was not apparent. This may be due to the complex interactions of the vertical and horizontal domains over space and time and especially due to human impacts^58^. Processes that affect soil quality are ploughing and uniform land management over longer time periods^58,59^ as they homogenize soil properties in the horizontal domain, at least over the size of agricultural fields as well as in the vertical domain, reflected in the ploughing depth. This effect is more pronounced on soil properties in the upper depth increments compared to the lower depth intervals. Consequently, the intense agricultural land use and other human activities may induce disruption of the steady-state soil processes in the upper depth increments^58,60^.

The increase of R^2^ of the additive machine learning model sequences and the decrease of R^2^ of the subtracting model sequences showed that the models improved with the multi-scale approach. This is due to the relation of CEC and θ_FC_ (with their texture and pH components) as well as pH to the large-scale subregional covariates that are functioning as proxies for the parent material (*p*), climate or other large scale translocation drivers as reported by Behrens et al.^17,18,20^: the higher octaves mainly reflect the geological properties and the geomorphic signature^61^ of the three-part study area since they allow for a spatial segmentation of the landscape units by aggregation due to scaling^18,62,63^. Therefore, the shape of the landscape, which is determined by the geological settings, is used inversely to separate the geological zones (Fig. 4).

The increase of R^2^ of the additive model sequences started at different scales. This means that for the different soil properties and each sampling increment, different scales are relevant in the study area and supports our hypothesis. The modelling sequences for topsoil increments tended to increase with intermediate octaves, whereas increases for subsoil increments were invariant. These differences in the additive model sequences can be explained regarding the state factor equation and the universal model of spatial variation. On the small scales the influence of the terrain covariates (*r*) on soil formation processes in topsoil is small, whereas the influence on soil processes in subsoil is larger. This may be related to the influence of catenary soil forming processes as erosion, sediment transport, and allocation^19,64^, which is active on intermediate scales (160–1280 m) and related to slope length. In contrast, subsoil is not influenced by recent erosion processes but by the geological and geomorphological history of the landscapes, incorporated into the model with the increasing Gaussian scale space as proxy data as explained above. The large-scale nature of the highest octaves also reflects the spatial context, i.e. the universal model of spatial variation, and the segmented study area that is, however, not limited to the geomorphic signature as described above, but also comprises land use types that can be differentiated on the supraregional level and that are related to the geomorphic setting (predominantly Dehesa and olive plantations in the mountain range, citrus plantations and irrigation-intensive crops at the floodplain and its proximity, and olive plantations and non-irrigated cereals at the Neogene terraces). The importance of the large-scale covariates in the modelling, thus, also reflects the human activities and their impact on soil formation through intensive agriculture as the accessibility for agricultural machines and the construction of irrigation facilities is affected by the geomorphology. This impact can either occur through erosion, sediment transport, and reallocation on the intermediate scale with catenary processes, but also through the different cultivated crops, irrigation, fertilization, and land use change over time that is closely related to socio-economic and political factors^65^.

The scaled terrain covariates did not contain additional relevant information for the model sequences for CEC and pH at 70–100 cm. Maybe further relevant information lies beyond the range recommended by the variogram. In general, Fig. 4 does not show a plateau in the R^2^ at larger scales, which was found in previous studies^8,16,17,20^. This indicates, that either additional larger octaves or predictors such as Euclidean distance transforms should be tested to increase modelling accuracies. It is recommended to increase the scales as far as possible with respect to interpretability and domain knowledge. Modelling beyond this maximum scale falls beyond the so-called information horizon^53^ and is only recommended when interpretability is not the objective.

To identify the effect of smallest relevant scales we used subtractive model sequences. The R^2^ of the subtractive model sequences decreased with the 6th octave and below for the soil quality indicators and for the 3rd–4th octave for topsoil and 6–7th octave for subsoil. Smaller scales did not improve the model performance, since they comprised irrelevant information or noise. Thus, they were not relevant for the individual models. This is related to the effective scales of the physical and chemical processes that are relevant for soil formation. One reason might be that the minimum scales of our input data required to achieve the most accurate model results are coarser than the original (finest) scale of the selected environmental covariates^18^. Regarding the agricultural use of the area and the size of the agricultural fields of a few hundred meters, the minimum range of multi-scale covariates, i.e., 80 m (4th octave), also may be linked to homogenization of soil with ploughing, fertilisation, and irrigation as mentioned above. Thus, the management practices may have disrupted steady-state soil processes on the small to intermediate scales^58,60^. Subsequently, the relevant scales were not only linked to small and large-scale geomorphologic patterns, but also to the physical, chemical, and biotic factors of farming^58^, which reshaped the landscape and may have blurred the scales reflecting the natural pattern of the landscape.

A direct comparison of the multi-scale model sequences with the empirical variograms is limited, since most of the relevant covariates have a spatial support that is smaller than the first lag of the variogram with a spatial support of 1500 m which corresponds to the 8th octave. Consequently, the multi-scale approach can account for soil forming processes on smaller scales than the variogram with automatically calculated lag distances. More research on this topic is necessary. However, even if we cannot quantify this effect, the better prediction results underpin the great potential of multi-scale modelling to predict soil processes and properties in landscapes that are both diverse in terms of their natural conditions and in terms of land use and land management.

In summary, since the different soil properties as well as the different soil depths showed varying relevant scales, we suggest that 3D soil models may require different sets of multi-scale environmental covariates to account for different soil forming processes of the state factor equation. Further, there may be landscapes where models for a certain soil depth do not benefit from multi-scale contextual information, because human activity may interfere with steady-state soil processes. An example are models for topsoil using satellite data that typically only reflect the upper centimetres of the Earth surface^24^.

Thus, disentangling natural and human signals over scales with multi-scale contextual modelling of soil processes and properties can pave the way from soil science to many other fields of research. Disciplines that are related to landscape development, e.g. (pedo-)archaeology, archaeobiology, geology, and climatology, benefit from the better understanding of spatial scales to reconstruct land use practices^66,67^ and paleo-climate conditions^13^. Since time can also be interpreted via spatial scales and is part of the state factor equation, the space-for-time substitution^68^ concept in geosciences and ecology^69^ that focuses on landscape development should incorporate scale in spatial analysis.

## Conclusion

There are two fundamental paradigms in digital soil mapping: the state factor equation with structural dependencies of soil formation and the universal model of spatial variation with spatial dependencies of soil formation. The multi-scale contextual spatial modelling approach is suitable to incorporate both structural and spatial dependencies into machine learning models. We hypothesised that different spatial scales may be relevant for different soil depths and interpreted how the differences may be related to the spatial context and soil formation.

We found:

- Different spatial scales are relevant in the horizontal (soil properties) and the vertical (soil depth) domain, and
- The relevant scales can be linked to the state factor equation and its factors relief (*r*), parent material (*p*) and organisms (*o*), i.e., land use and human impact.

These examples show, that pedologic and (physical and human) geographic domain knowledge is important to link the factors of the state factor equation and to interpret the relations to potential soil forming processes in different soil depths. Thus, we suggest using multi-scale environmental covariates with different spatial support to account for the specific soil forming processes in specific soil depths when modelling soil properties in 3D.

## Supplementary Information

Supplementary Information.

## Data Availability

The original terrain data is published under the CC-BY 4.0 license by Centro Nacional de Information Geográfica (CNIG) of the Spanish government; last accessed March 31st 2020 (https://doi.org/10.7419/162.09.2020). The soil data and the processed terrain data is published at PANGAEA (https://doi.pangaea.de/10.1594/PANGAEA.938522 and https://doi.pangaea.de/10.1594/PANGAEA.938774).
